# Maternal Sepsis and associated factors: A multi-central study from two tertiary care hospitals of South Punjab, Pakistan

**DOI:** 10.12669/pjms.41.1.10423

**Published:** 2025-01

**Authors:** Rashida Parveen, Hajra Sultana, Sadia Nazir

**Affiliations:** 1 Rashida Parveen, FCPS Associate Professor / HOD Obstetrics & Gynaecology Department, DG Khan Medical College, DG Khan, Pakistan; 2 Hajra Sultana, FCPS Associate Professor, Obstetrics and Gynaecology Department, Nishtar Medical University, Multan, Pakistan; 3 Sadia Nazir, FCPS Assistant Professor, Obstetrics and Gynaecology Department, DG Khan Medical College, DG Khan, Pakistan

**Keywords:** Blood culture, *E. coli*, Gestational age, Mortality, Sepsis

## Abstract

**Objective:**

To determine the risk factors and outcomes of maternal sepsis.

**Methods:**

This case-control study was performed at the departments of Obstetrics & Gynecology, Nishtar Hospital, Multan, and Ghazi Khan Hospital, Dera Ghazi Khan, Pakistan, from June 2023 to May 2024. Cases were comprised of females aged 18-45 years diagnosed with maternal sepsis, and admitted during the study period. Controls were randomly selected females reporting during the study period and undergoing delivery. Sepsis was labeled on the basis of positive blood culture report. Crude and adjusted odds ratio with 95% confidence interval were reported regarding various risk factors of maternal sepsis as well as maternal and fetal outcomes taking p<0.05 as significant. Mortality was noted from the onset of labor until seven days postpartum.

**Results:**

In a total of 74 women (37 in each group), the mean age and, gestational age were 30.64±5.12 years, and 36.19±1.84 weeks, respectively. Multivariate binary logistic regression showed that gestational age below 37 weeks (AOR: 5.22; 95% CI: 1.35-19.67; p=0.015), unbooked cases (AOR: 5.34; 95% CI: 1.19-24.2; p=0.029), and anemia (AOR: 8.13; 95% CI: 1.05-63.10; p=0.045) were significant predictors of maternal sepsis. *E. coli* was the most common etiological agent among cases, affecting 14 (37.8%) cases. Mortality was significantly high among females with maternal sepsis versus those without maternal sepsis (32.4% vs. 2.7%, p=0.008).

**Conclusion:**

Gestational age below 37 weeks, lack of antenatal booking, and anemia were found to be significant predictors of maternal sepsis. *E. coli* was the most common pathogen identified. High mortality rate in maternal sepsis highlights the need for early identification, effective management, and close monitoring to reduce maternal mortality from sepsis.

## INTRODUCTION

Maternal sepsis is a serious condition that results from organ dysfunction due to infection during pregnancy, childbirth, post-abortion, or the postpartum period.^1^ Maternal sepsis is considered to be an important cause behind maternal mortality and morbidity, accounting for an annual burden of 10.7% maternal mortality globally.^2^ Women in low-resource countries are disproportionately affected by sepsis due to insufficient diagnostic tools, inadequate management, a shortage of skilled birth attendants, poor infection prevention and control practices, and lack of access to clean water and sanitation.^3^ While high-income countries have made significant strides in reducing maternal sepsis-related mortality and morbidity, low-income countries face challenges in implementing WHO-recommended interventions.^4^

Diagnosing sepsis during pregnancy is particularly difficult because physiological changes and obstetric interventions can obscure typical clinical signs. In low-income countries, the absence of effective diagnostic and management protocols delays sepsis diagnosis, leading to higher rates of septic shock, critical care admissions, and the need for respiratory support.^5^ The differences in the management of maternal sepsis were exhibited by the one week prospective GLOSS study, which evaluated maternal infection rates and care practices in 713 facilities across 52 countries.^6^ This study highlighted the importance of adopting evidence-based practices to prevent, diagnose early, and manage maternal infections, with the goal of reducing morbidity and mortality in healthcare settings. Zhong et al from China noted that gestational diabetes was present in 27.3% cases of postpartum sepsis versus 15.3% without sepsis.^7^ It is important to assess the impact of potential contributors to maternal sepsis at resources limited centers like us so this study was planned. This study was conducted to determine the risk factors and outcomes of maternal sepsis.

## METHODS

This case-control study was performed at the departments of Obstetrics & Gynecology, Nishtar Hospital, Multan, and Ghazi Khan Hospital, Dera Ghazi Khan, Pakistan, from June 2023 to May 2024. Sample size was calculated to be 74 (37 in each group), taking the proportion of cesarean section as 75.8% among women with maternal sepsis versus 39.0% without maternal sepsis,^7^ with 95% confidence level, and 80% power.

### Ethical Approval:

It was obtained from the Institutional Ethical Review Board of Nishtar Medical University”, Multan (letter number: 7077, dated June 15, 2024), and “Ethical Review Committee of DG Khan Medical College, Dera Ghazi Khan” (letter number: 116, dated May 31, 2024).

### Inclusion & Exclusion criteria:

Females aged 18-45 years with maternal sepsis. Women having acute pulmonary or amniotic fluid embolism, chronic liver disease, or those with autoimmune conditions were not included. For cases, non-probability, consecutive sample technique was be employed. For controls, simple random sampling technique was applied. Controls were randomly selected females reporting during the study period and undergoing delivery. Sepsis was labeled on the basis of positive blood culture report and defined as infection plus systemic manifestation of infection.^8^

Written as well as informed consents were taken from all study participants informing them the aims and objectives of this study. At the time of enrollment, demographic and clinical findings were recorded. Period of sepsis onset was described as antepartum, intrapartum, or postpartum. Data like mode of delivery, maternal complications, intensive care unit (ICU) admissions, and maternal as well as neonatal outcomes were noted. APACHE-II scores on the first day of ICU admission were recorded to evaluate the severity of illness. Gestational diabetes was defined as the fasting 75g oral glucose tolerance test during pregnancy and diagnosed if fasting glucose ≥5.8, 1-hour glucose ≥10 mmol/L, or two hours’ glucose ≥11.1 mmol/L.^7^ Preeclampsia was defined as the confirmation of hypertension arising after 20 weeks of gestation on two or more occasions and one or more of the organ/system features related to the mother and/or fetus.^7^ Anemia was labeled as hemoglobin below 10 g/dl.^9^ A special proforma is designed to record all study data.

### Statistical Analysis:

Data were entered and analyzed using IBM-SPSS Statistics, version 26.0. Qualitative data were presented in the form of frequency and percentages. Quantitative variables were shown as mean and standard deviation. Chi-square test was applied to compare categorical data. Crude and adjusted odds ratio with 95% confidence interval were reported regarding various risk factors of maternal sepsis as well as maternal and fetal outcomes taking p<0.05 as significant.

## RESULTS

In a total of 74 women, the mean age and, gestational age was 30.64±5.12 years (ranging between 20-42 years), and 36.19±1.84 weeks (ranging between 32-39 weeks, respectively. There were 40 (54.1%) women who were aged between 18-30 years. Forty-four (59.5%) women were prim parous. The mean BMI was 27.16±2.38 (kg/m^2^). Gestational diabetes, and pre-eclampsia were noted among 12 (16.2%), and 13 (17.6%) women, respectively. Table-I is showing comparison of baseline characteristics among cases and controls. There were 49 (66.2%) women who were unbooked. Among cases, the mean APACHE-II score was 12.53±2.05. Univariate analysis revealed gestational age (p<0.001), gestational diabetes (p0.022), pre-eclampsia (p=0.042), booking status (p<0.001), and anemia (p=0.005) to be significantly associated with maternal sepsis. Multivariate binary logistic regression showed that gestational age below 37 weeks (AOR: 5.22; 95% CI: 1.35-19.67; p=0.015), unbooked cases (AOR: 5.34; 95% CI: 1.19-24.2; p=0.029), and anemia (AOR: 8.13; 95% CI: 1.05-63.10; p=0.045) were significant predictors of maternal sepsis.

**Table-I:** Baseline characteristics of Women in both study groups (N=74).

Characteristics			Cases (n=37)	Controls (n=37)	COR with 95% CI (Lower-Upper)	P-value	AOR with 95% CI (Lower-Upper)	P-value
Age (years)	18-30	18 (48.6%)		22 (59.5%)	0.65 (0.26-1.62)	0.352	0.422 (0.89-2.00)	0.277
	31-45	19 (51.4%)		15 (40.5%)	Reference		Reference
Residence	Rural	20 (54.1%)		16 (43.2%)	1.54 (0.62-3.86)	0.353	1.04 (0.29-3.79)	0.954
	Urban	17 (45.9%)		21 (56.8%)	Reference		References
Parity	Primiparous	23 (62.2%)		21 (56.8%)	1.25 (0.49-3.17)	0.636	1.69 (0.35-8.04)	0.511
	Multiparous	14 (37.8%)		16 (43.2%)	Reference		Reference
Gestational age (weeks)	<37	26 (70.3%)		9 (24.3%)	7.35 (2.62-20.60)	<0.001	5.22 (1.35-19.67)	0.015
	≥37	11 (29.7%)		28 (75.7%)	Reference		Reference	
Body mass index (kg/m^2^)	<30	29 (78.4%)		32 (86.5%)	Reference	0.363	Reference	
	≥30	8 (21.6%)		5 (13.5%)	1.77 (0.52-6.01)	1.71 (0.35-8.38)	0.506
Gestational diabetes			10 (27.0%)	2 (5.4%)	6.49 (1.31-32.07)	0.022	9.26 (0.82-104.47)	0.072
Pre-eclampsia			10 (27.0%)	3 (8.1%)	4.20 (1.05-16.78)	0.042	1.60 (0.28-9.27)	0.602
Booking status	Booked	5(13.5%)		20 (54.1%)	Reference	<0.001	Reference	
	Unbooked	32 (86.5%)		17 (45.9%)	3.66 (1.36-9.86)		5.34 (1.19-24.21)	0.029
Anemia			35 (94.6%)	24 (64.9%)	9.48 (1.96-45.87)	0.005	8.13 (1.05-63.10)	0.045

Evaluation about the period of sepsis onset among cases revealed that 11 (29.7%) women experienced sepsis during antepartum period, 10 (27.0%) intrapartum, and 13 (35.1%) during postpartum phase, while in remaining 3 (8.1%), we were unable to label the onset of sepsis period. *E.coli* was the most common etiological agent among cases, affecting 14 (37.8%) cases, whereas, MRSA, Streptococcus sp., and klebsiella pneumoniae were the other most frequently identified etiological agents, in 9 (12.2%), 6 (8.1%), and 2 (2.7%) cases, respectively.

Overall, mortality was reported in 13 (17.6%) mothers. Fetal outcomes were reported to be live-birth, still-birth, and abortion were documented in 61 (82.4%), 8 (10.8%), and 5 (6.8%), respectively. Univariate analysis revealed mode of delivery (p=0.009), maternal ICU admission (p<0.001), maternal death (p=0.008), and still-birth (p=0.041) to be significantly associated with maternal sepsis. Multivariate binary logistic regression revealed maternal ICU admission (AOR: 10.31; 95% CI: 3.09-34.40; p<0.001) to be significantly linked with maternal sepsis. Table-II is showing details about the maternal and fetal outcomes.

**Table-II:** Maternal and fetal outcomes in both study groups (N=74).

Outcome variables		Cases (n=37)	Controls (n=37)	COR with 95% CI (Lower-Upper)	P-value	AOR with 95% CI (Lower-Upper)	P-value
Mode of delivery	Vaginal delivery	8 (21.6%)	19 (51.4%)	Reference	0.009	Reference	0.269
	Cesarean section	29 (78.4%)	18 (48.6%)	3.83 (1.39-10.55)		2.03 (0.58-7.12)
Maternal ICU admissions	Yes	28 (75.7%)	7 (18.9%)	13.33 (4.37-40.62)	<0.001	10.31 (3.09-34.40	<0.001
	No	9 (24.3%)	30 (81.8%)	Reference		Reference
Maternal outcomes	Survived	25 (67.6%)	36 (97.3%)	Reference	0.008	Reference	0.057
	Died	12 (32.4%)	1 (2.7%)	17.28 (2.11-141.51)		11.07 (0.93-132.29)
Fetal outcomes	Abortion	4 (10.8%)	1 (2.7%)	5.39 (0.57-51.05)	0.142	1.73 (0.10-29.41)	0.705
	Still-birth	7 (18.9%)	1 (2.7%)	9.42 (1.09-81.37)	0.041	2.22 (0.14-36.25)	0.575
	Live-birth	26 (70.3%)	35 (94.6%)	Reference	-	Reference	-

## DISCUSSION

Our analysis identified that gestational age below 37 weeks (AOR 5.22, p=0.015), unbooked cases (AOR 5.34, p=0.029), and anemia (AOR 8.13, p=0.045) were strong predictors of maternal sepsis. A local study done by Bakhtawar et al documented that preterm delivery (95% CI: 1.9–20.2), lower abdominal pain (95% CI: 1.2–3.4), vaginal discharge (95% CI: 3.0–20.2), and SpO2 < 93% (95%: CI 4.8–37.1) had significant association with maternal sepsis.^10^ Literature has also pointed out hemorrhage, lacerations, frequent vaginal evaluations, and mode of delivery as important predictors of maternal sepsis.^11^,^12^

A study done by Zhong et al described pre-eclampsia to be independent predictor of maternal sepsis. In this study, univariate analysis revealed pre-eclampsia to have significant association with the existence of maternal sepsis (COR: 4.20; 95CI: 1.05-16.78, p=0.042) but in multivariate binary logistic regression, final model did not reveal pre-eclampsia to be a significant predictor of maternal sepsis (AOR: 1.60; 95CI: 0.28-9.27; p=0.602).^7^ Acosta et al from Scottland, revealed that relatively younger age, and cesarean section were significant predictors of maternal sepsis.^13^

All these studies show that spectrum and possible predictors of maternal sepsis vary between different populations so it is imperative to analyze these insights periodically. Difference in antenatal care, socio-demographic characteristics, ethnic and family behaviors might be playing important role behind maternal sepsis and we need more studies to evaluate these aspects of maternal sepsis. We noted that most of the women (35.1%) women experienced sepsis during postpartum period, 29.7% during antepartum period, and 27.0% during intrapartum period. These findings are consistent with what was noted by Shivanada et al from India where they stated that 50% of maternal sepsis cases were thought to have experienced sepsis during postpartum period.^14^

Another study reported similar finding where females are thought to be most vulnerable to maternal sepsis during postpartum period.^15^ Kankuri et al. and colleagues showed that cesarean delivery raises the risk of maternal sepsis as 2.6 times during antepartum sepsis, whereas overall risk of maternal sepsis was 3.2 fold higher during postpartum phase.^16^ *E. coli* was the most common bacterial agent (37.8%) among maternal sepsis women. *E. coli* has general been identified as the most frequent bacterial isolates in maternal sepsis globally.^13^,^17^-^19^ Local antimicrobial susceptibility and resistance patterns must be analyzed to keep track regarding the most viable options against most common bacterial isolates is different populations.

The present research revealed that mortality was significantly high among females with maternal sepsis versus those without maternal sepsis (32.4% vs. 2.7%, p=0.008). Regional data about maternal mortality rates in sepsis as high as 80% have been reported.^19^ Timely identifications, aggressive management and active monitoring are keys to improve outcomes in maternal sepsis.^20^ The study was conducted at two of the leading tertiary healthcare facilities of South Punjab Pakistan that receives cases from all over South Punjab regions of Punjab Province, Pakistan, that increases the generalisability of this research.

Sepsis is increasingly recognized as a significant health issue with high mortality rates. The scarcity of comprehensive data about the incidence, epidemiology, and outcomes of maternal sepsis, particularly in lower-income and lower-middle-income countries, complicates efforts to compare and estimate the overall burden.^21^ This data gap highlights the need for further research in this area to establish a foundation for future studies. The present study was an effort to explore factors associated with maternal sepsis.

### Limitations:

We were unable to evaluate factors associated with the development of maternal sepsis. Antimicrobial susceptibility and resistance patterns were not recorded. Data about socioeconomic status, choice of antimicrobials and details about intravenous fluids were not the part of data collection.

## CONCLUSION

Gestational age below 37 weeks, lack of antenatal booking, and anemia were found to be significant predictors of maternal sepsis. *E. coli* was the most common pathogen identified. The mortality rate among women with maternal sepsis highlights the need for early identification, effective management, and close monitoring to reduce maternal mortality from sepsis

### Authors contribution:

**RP:** Data collection and synthesis, drafting, Literature search, critical review, responsible for data’s integrity, approved the final draft for publication.

**HS:** Data analysis, drafting, proof reading, critical revisions, approval the final draft for publication.

**SN:** Data collection and synthesis, drafting, proof reading, critical review, approved the draft for publication.
